# Cumulative effects of human footprint, natural features and predation risk best predict seasonal resource selection by white-tailed deer

**DOI:** 10.1038/s41598-022-05018-z

**Published:** 2022-01-20

**Authors:** Siobhan Darlington, Andrew Ladle, A. Cole Burton, John P. Volpe, Jason T. Fisher

**Affiliations:** 1 Department of Biology, University of British Columbia Okanagan Campus, 1177 Research Road, Kelowna, BC V1V 1V7 Canada; 2 School of Environmental Studies, University of Victoria, PO Box 1700 STN CSC, Victoria, BC V8W 2Y2 Canada; 3 Department of Forest Resources Management, University of British Columbia, Forest Sciences Centre 2045 – 2424 Main Mall, Vancouver, BC V6T 1Z4 Canada

**Keywords:** Behavioural ecology, Ecological modelling, Invasive species, Population dynamics, Environmental impact

## Abstract

Land modified for human use alters matrix shape and composition and is a leading contributor to global biodiversity loss. It can also play a key role in facilitating range expansion and ecosystem invasion by anthrophilic species, as it can alter food abundance and distribution while also influencing predation risk; the relative roles of these processes are key to habitat selection theory. We researched these relative influences by examining human footprint, natural habitat, and predator occurrence on seasonal habitat selection by range-expanding boreal white-tailed deer (*Odocoileus virginianus*) in the oil sands of western Canada. We hypothesized that polygonal industrial features (e.g. cutblocks, well sites) drive deer distributions as sources of early seral forage, while linear features (*e.g.* roads, trails, and seismic lines) and habitat associated with predators are avoided by deer. We developed seasonal 2nd -order resource selection models from three years of deer GPS-telemetry data, a camera-trap-based model of predator occurrence, and landscape spatial data to weigh evidence for six competing hypotheses. Deer habitat selection was best explained by the combination of polygonal and linear features, intact deciduous forest, and wolf (*Canis lupus*) occurrence. Deer strongly selected for linear features such as roads and trails, despite a potential increased risk of wolf encounters. Linear features may attract deer by providing high density forage opportunity in heavily exploited landscapes, facilitating expansion into the boreal north.

## Introduction

Habitat loss from the conversion of mature forests for human use is the most intensive form of human disturbance to forest species^1^, but the deleterious effects of fragmentation of otherwise intact forests by road networks and other linear features have been increasingly recognized^2–4^. Imposing anthropogenic landscape features of different shapes and patch composition onto a forest matrix can alter biodiversity indirectly by modifying community structure, shifting species distributions^5–7^ and influencing animal behaviour^8^. For any given species, landscape changes can manifest as altered abundance and distribution of food resources, as well as altered predation risk – two key components of resource selection for any prey species^9,10^.

Anthropogenic land-use change often creates suitable conditions for invasive and early seral vegetation that support herbivores that are better adapted to exploit anthropogenic landscapes^11,12^. Where herbivores capitalize upon novel sources of available forage^13^, land-use change may lead to expanding distributions. Range-expanding species can be considered invasive in that they negatively impact biodiversity and ecosystem function directly through increased competition and predation^14^, and indirectly through changes in disturbance regimes, nutrient levels, and micro-climate^15,16^.

In the western Nearctic boreal forest, extensive forest harvesting and petroleum extraction have altered landscape shape and composition^17^ outside the range of natural variability^18^. These disturbance types cumulatively alter species distributions^5,19^ and favour generalist species. In particular, white-tailed deer (*Odocoileus virginianus,* hereby referred to as ‘deer’) have thrived in this rapidly changing landscape as evidenced by the expansion of their northern range limit over the past fifty years, with populations increasing in abundance in areas of high human disturbance^20–22^. Historically limited by deep snow, poor quality forage, and cold temperatures, deer are now one of the most pervasive ungulates in western Canada’s boreal forest^5,19,23^. Deer range expansion is an indirect cause of decline for native subspecies of woodland caribou (*Rangifer tarandus caribou*)^20,24^, acting as apparent competitors by inflating the population size of their shared predator, wolves (*Canis lupus*), thereby increasing predation on caribou^25^.

The role of human land-use in sustaining boreal deer populations is attributed to early seral vegetation from forestry cutblocks^18^, petroleum extraction, and transportation networks^26,27^. Focus has been on polygonal features that create patches of disturbance, such as well pads and forestry cutblocks. There has been less focus on the widespread, high-density linear features (e.g. roads, seismic lines^17,28^) as sources of forage or predation risk^24^. While the response of other species, such as wolves (*Canis lupus*), to linear features is well known^26,29^ less is known about the role of linear features on ungulate habitat selection in the western boreal forest^30,31^ though these constitute one of the most intensive and extensive anthropogenic disturbances on western boreal landscapes^28^.

In eastern North America deer select roadside verges^32^ and deciduous stands, such that deer abundance increases with road density and forage opportunity^33,34^. However, the cumulative effects of two different disturbance forms—polygonal early-successional patches and linear features – on deer distribution and reproduction are a key emerging pattern^27^. For example, in western North America, deer reproductive success increases in areas with intensive resource extraction^35^. The behavioural component of these responses remains unclear.

More importantly, few studies have examined deer response to cumulative landscape disturbance in the context of predation risk, which is critical for most prey species. The theoretical underpinning of the interaction between anthropogenic landscape change and predation risk lies in optimal foraging theory, which predicts that animals will attempt to maximize energy gain per unit cost; hence deer will seek to maximize foraging opportunities while minimizing their exposure to temperature extremes, predators, and other threats^36^. Perceived predation risk results in anti-predator avoidance behaviour both temporally and spatially^37,38^ and can manifest as indirect predation risk when prey avoid landscape features used by predators^38^. Energetic trade-offs between forage acquisition and predation risk have been observed in deer^38^ and other ungulates^38^ including within the context of oil and gas development^8^. Deer energetic requirements in the boreal shield vary with biological and geographic seasonality, peaking during winter when movement and foraging are limited by deep snow^20,39,40^, compared to autumn when males compete for mates in the rut, or in spring when females give birth^41^. Understanding anti-predator response in prey habitat selection studies may better explain the impacts of landscape disturbance on prey distributions and the mechanisms of range expansion; it can focus restoration and management efforts to mitigate changes to spatial predator–prey processes and limit resulting negative impacts to wildlife species, and so remains a key pursuit for landscape ecologists and conservationists.

We ask whether linear and polygonal anthropogenic features, predation risk, or natural habitat best explain seasonal habitat selection of range-expanding Nearctic deer populations. Previous studies on deer near the limits of their northern range have used aerial surveys^25^, snow tracking^23^, and camera detections^42^ to quantify the relative importance of landscape disturbance to deer, but behavioural response requires individual-specific, high-density location data. We use high-frequency GPS collar data to examine seasonal habitat selection by deer at the level of their home range, where landscape disturbance is relevant to range expansion in the population.

We weigh support for six competing hypotheses to explain variability in deer habitat selection: (1) forage acquisition hypothesis, whereby deer select polygonal disturbances due to increased resource subsidies; (2) indirect predation risk hypothesis, whereby deer avoid linear features as heavily-used predator travel corridors; (3) predator-frequency avoidance hypothesis, whereby deer avoid high-use areas measured as predicted monthly occurrence of wolves and black bears; (4) null hypothesis, whereby deer select natural habitats not necessarily associated with subsidy or risk, delineated by different forest and vegetation types; (5) human footprint hypothesis, where deer make trade-offs with avoidance of industrial linear and selection for polygonal features; and (6) cumulative effects hypothesis, whereby deer respond to the combined effects of all natural and anthropogenic variables.

## Methods

### Study area

**Figure 1:**
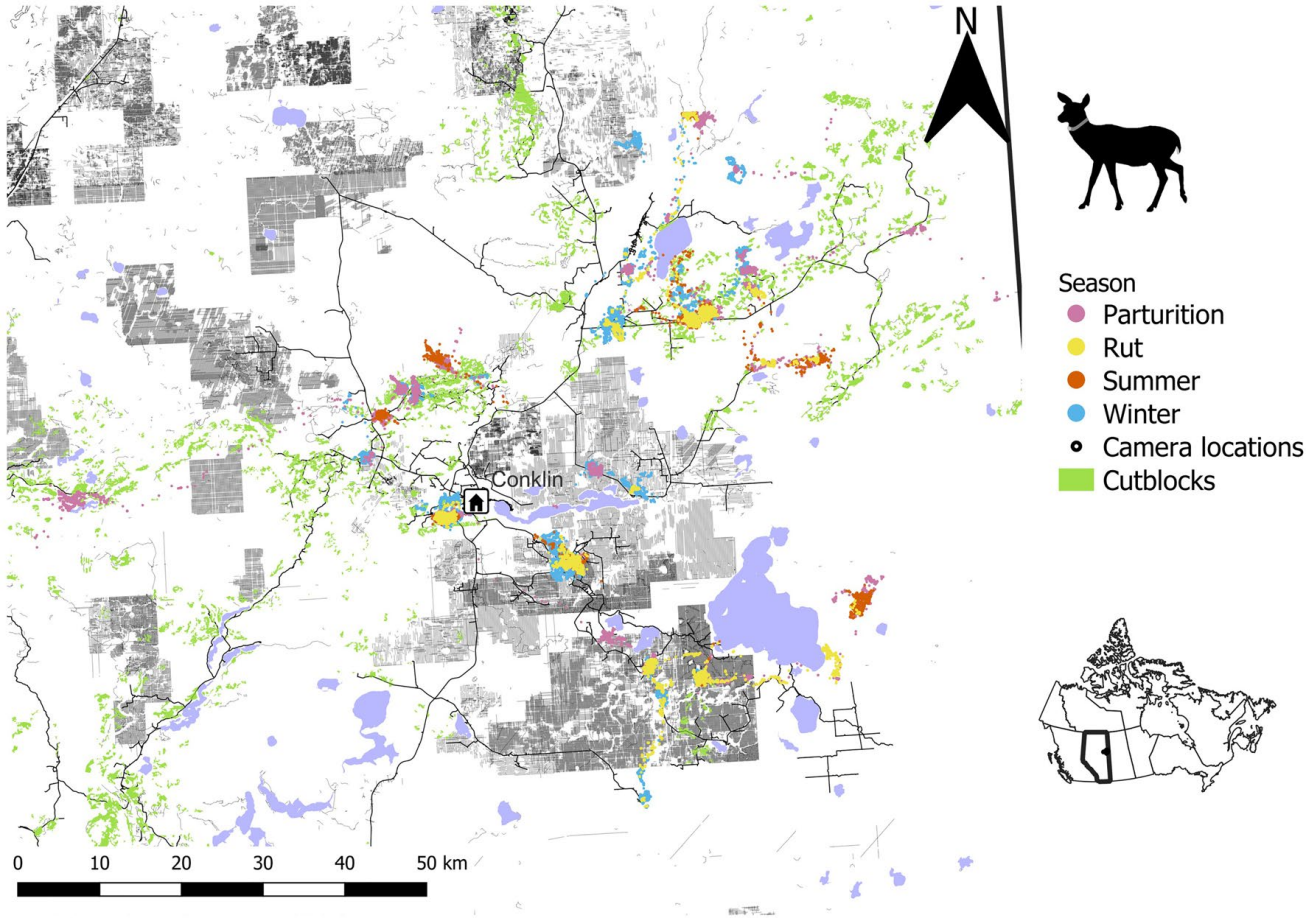
Seasonal white-tailed deer telemetry data collected in the Christina Lake study area near Conklin, Alberta encompassing 3500 km^2^ from 2012 to 2014. The area is extensively developed from forest harvesting and petroleum extraction, with features such as 3D seismic lines, legacy seismic lines, and roads appearing in as grey linear features and 0–10-year-old cutblocks as green polygons. Wetlands appear in purple.

Our sampling frame is the western boreal forest of Canada. Our study area encompasses 3500 km^2^ of mixed-wood boreal forest in northeast Alberta intersecting the ranges of the Cold Lake and the East-side Athabasca River caribou herds^43^ (Fig. 1) and represents a high industrial disturbance portion of the frame. Polygonal features and linearization are spread across a gradient of disturbance from oil and gas development and forestry^17^. This fragmented, grid-like landscape of cleared forest is slow to regenerate^28^, creating a wide-spread source of early seral forage^18^ that benefit deer and are easily navigable by predators^26,29^. Intact natural vegetation is a heterogeneous mosaic of mixed-wood boreal forest dominated by black spruce (*Picea mariana*) and trembling aspen (*Populus tremuloides*) in lowland and upland deciduous stands with intersecting bogs, lakes, and rivers.

### Deer telemetry

Thirty-eight female deer were captured and collared in the winters of 2012, 2013, and 2014 in accordance with animal care protocols and permitted by Alberta Environment and Parks (Permits 49365 and 48602). All methods are reported in accordance with ARRIVE guidelines^44^. Deer were captured using clover traps which minimize deer stress relative to other modes of capture^45^. Individuals were fitted with LOTEK Iridium Track M 3D telemetry collars programmed to record locations at 2-h fixed intervals. Individuals with < 200 telemetry locations were removed (n = 2) to avoid including individuals with limited home-range coverage, resulting in 36 deer included within the analysis. We defined biological seasons for deer according to their life history stages and geography. Winter occurs from January 1st-April 30th, parturition from May 1st to June 30th, summer from July 1st to September 30th and rut from October 1st to December 31st^20^.

### Estimating predation risk from camera trap data

Predation risk can be characterized as *risky times* or *risky places*^46^. Risky times are indicated by direct proximal predation risk by co-occurring predators; risky places are those where probability of predator encounter is high (but not necessarily occupied by a predator in a given time period^46^). Here we considered ‘risky places’ as those with high use by boreal deer primary predators: wolves (*Canis lupus*) and black bears (*Ursus americanus)*^40,47,48^*.* We did not have a direct measure of predation outcomes (e.g. mortalities attributed to predation, concurrently collared predators) so we used the probability of monthly occurrence of a predator at a site (hereafter occurrence frequency) as an indirect measure of risk, which we derived from a concurrent camera-trap survey in the study area^5^. The camera array of unbaited infra-red Reconyx PC900 Hyperfire remote digital camera was deployed using a stratified random design (see Fisher and Burton, 2018 for more details) at 62 sites from October 2011 to October 2014. Cameras were placed at least 2 km apart and the nearest camera to a road was 50 m with road density used as a model parameter. Predator-distribution models were developed by Fisher and Burton (2018); we used beta coefficients from the best-supported wolf and black bear models of occurrence frequency (Table S1) to extrapolate occurrence frequency for each predator across the study area using ArcMap 10.5 (Fig. S1). We assumed that model-estimated probability of monthly occurrence of each predator species is linearly related to the likelihood of collared deer encountering those predators, which we considered as a measure of predation risk^38^.

### Landscape covariates

To examine deer response to natural habitat characteristics and anthropogenic forms of landscape disturbance relating to forage acquisition and predation risk, we used natural and anthropogenic land cover data quantified in ArcMap 10.5 (Table 1). Forest cover—percent crown closure of dominant overstorey species—were obtained from the Alberta Vegetation Index (AVI^49^). Anthropogenic landscape features were derived from Alberta Biodiversity Monitoring Institute (ABMI) human footprint maps (ambi.ca), with contemporary additional industrial features supplied by the ABMI Caribou Monitoring Unit (cmu.abmi.ca).

**Table 1:** Core hypotheses and corresponding landscape variables used to quantify natural landscape features from the Alberta Vegetation Inventory (AVI), and anthropogenic landscape features from two sources: industrial linear features from the Alberta Biodiversity Monitoring Institute (ABMI) Caribou Monitoring Unit (CMU) updated to 2012, and industrial block features from the ABMI human footprint project updated from 2010.

Hypothesis: Deer select for	Variable Name	Description	Source
Resource subsidy^a^	DistCutblock	Distance to (m) forest harvesting areas with mature trees removed and saplings regrowing from 2000 to 2010	ABMI 2010
	DistWellSites	Distance to (m) well pads deforested for in-situ oil extraction	
Linear Features^a^	DistSeismic	Distance to (m) traditional seismic petroleum exploration line *ca.* 7–10 m wide	ABMI CMU 2012
	Dist3DSeismic	Distance to (m) seismic petroleum exploration line *ca.* 1–3 m wide	
	DistPipe	Distance to (m) petroleum pipeline and grassy right of way	
	DistRail	Distance to (m) railway line and associated vegetated right of way	
	DistRoad	Distance to (m) hard surface road, Roads including vegetated verge, Unimproved (gravel) roads, truck trails, winter roads	
	DistTrail	Distance to (m) unimproved dirt track ca. 5–10 m wide navigable by off-highway vehicle or foot	
Natural Features	DistWetland	Distance to (m) open wetland including lakes, streams, and bogs	AVI
	PCT Aw^b^	Trembling aspen *Populus tremulodies*	
	PCT Bw	White birch *Betula papyrifera*	
	PCT Fb	Balsam fir *Abies balsamea*	
	PCT Lt	Tamarack *Larix laricina*	
	PCT Pb	Balsam poplar *Populus balsamifera*	
	PCT Pj	Jack pine *Pinus banksiana*	
	PCT Sb	Black spruce *Picea mariana*	
	PCT Sw	White spruce *Picea glauca*	
Indirect Predation Risk	Wolf GLM	Grey wolf probability of monthly occurrence extrapolated using 2250 m search radius and scaled from 0–1	Fisher and Burton 2018
	Bear GLM	Black bear probability of monthly occurrence extrapolated using 500 m search radius and scaled from 0–1	

All distance-to metrics are measured in metres (m).

^a^These are composite variables, we measured the distance to the closest of any of these features.

^b^PCT refers to the *percent* of the forest canopy overstorey dominated by this leading tree species. AVI data were created and provided by Alberta Environment and Sustainable Resource Development 2010. Provincial Human Footprint layers and 2012 linear features were provided by ABMI and University of Alberta, Integrated Landscape Management Lab.

### Seasonal resource selection functions

Resource selection functions (RSFs) characterize the probability of a resource unit being selected by an organism in a use-availability framework^50^. They are estimated by regressing used telemetry locations (1 s) against randomly generated available locations (0 s) within a defined domain of availability^51^. As our main interest related to habitat selection by a range-expanding ungulate, we were interested in second order selection (Johnson 1980); where individual deer selected their seasonal home ranges from within the surrounding available landscape. We therefore defined the domain of availability for each season as the buffered 100% minimum convex polygon (MCP) surrounding all used deer locations within the associated season. We calculated the mean radius of seasonal home-range areas and used these as buffer distances (winter = 2093 m, parturition = 4249 m, summer = 2172 m, rut = 3903 m) beyond each seasonal MCP; we randomly selected available locations, at a 1:1 used: available ratio, from these areas. Resource variables (Table 1) were extracted for each used and available location using the *raster* package^52^ in R 4.1.1^53^. To check that the available sample represented the available landscape, we identified deer with the smallest and largest number of used locations for each season. Next, we created ten bootstrapped available datasets, each consisting of two times the number of used locations randomly sampled from within the respective deer’s seasonal MCPs, extracted covariates, and visually compared the distribution of variables across bootstrapped available samples. Variable distributions sufficiently overlapped across the bootstrapped samples (Figure S2–S4), irrespective of deer sample size (our sampling unit).

We generated six RSF models (Table 2) for each of four biological seasons to examine the drivers of population-scale deer resource selection. We used logistic regression in a Generalized Linear Mixed-Effects Model (GLMM)^54^. As we had a number of deer that contained data across multiple years, we included “individual-year” as a random effect on the intercept to avoid potential pseudo-replication arising from the dependent nature of telemetry data sampled from individuals and years^55^. We log-transformed distance variables and standardized percent cover and predator occurrence covariates (mean = 0, s.d. = 1) and tested for multicollinearity^54^. We excluded collinear variables with r > 0.7 and a variance inflation factor (VIF) > 3^56^.

**Table 2:** White-tailed deer population scale Resource Selection Function (RSF) hypotheses and corresponding model sets and variables in northeastern Alberta. Each model set was tested across four seasons: winter, parturition, rut and summer to detect seasonal variation in selection. All distance variables were log-transformed.

Model set	Model variables	Hypotheses: Deer resource selection is best predicted by
Resource	DistCutBlock + DistWellSite	Distance to industrial block features as sources of early seral forage
Linear	DistRoad + DistSeismic + DistSeismic3D + DistPipe + DistTrail	Distance to industrial linear features as an indirect measure of predation risk
Predation risk	Wolf.GLM + Bear.GLM	Relative predator abundance or increased likelihood of encounters
Natural	PCT.Aw + PCT.Sb + PCT.Bw + PCT.Sw + PCT.Pb + PCT.Lt + PCT.Pj + PCT.Fb + DistWetland	Naturally occurring forage and canopy cover
Human Footprint	DistCutBlock + DistWellSite + DistRoad + DistSeismic + DistSeismic3D + DistPipe + DistTrail	The effects of all polygonal and linear industrial landuse
Cumulative	DistCutBlock + DistWellSite + DistRoad + DistSeismic + DistSeismic3D + DistPipe + DistTrail + Wolf.GLM + Bear.GLM + PCT.Aw + PCT.Sb + PCT.Bw + PCT.Sw + PCT.Pb + PCT.Lt + PCT.Pj + PCT.Fb + DistWetland	The cumulative effects of human footprint, natural habitat, and predation risk

Variable descriptions can be referenced in Table 1.

Models within each season were ranked using an information-theoretic approach based on Akaike Information Criteria corrected for small sample size (AICc) scores where the lowest AICc score reflects the most parsimonious model with the most deviance explained^57,58^. We used k-fold cross validation, whereby each individual’s data within corresponding seasons were split into k = 10 folds to validate each candidate model^51^ and each subset was evaluated using models trained with k – 9 alternative subsets.

### Ethics and permits

All animals were captured and processed under approved handling protocols by InnoTech Alberta’s Animal Care Committee and permitted by Alberta Environment and Parks (Permit #s 49365 and 48602). All methods are reported in accordance with ARRIVE guidelines.

## Results

### Telemetry data

A total of 99,148 telemetry locations were obtained over the three-year period with a mean of 2754 locations per individual (range: 254–12,236). These data were subset by season resulting in 46,722 locations in winter, 27,573 in parturition, 9,987 in summer, and 14,866 in rut.

### Seasonal drivers of deer habitat selection

**Figure 2:**
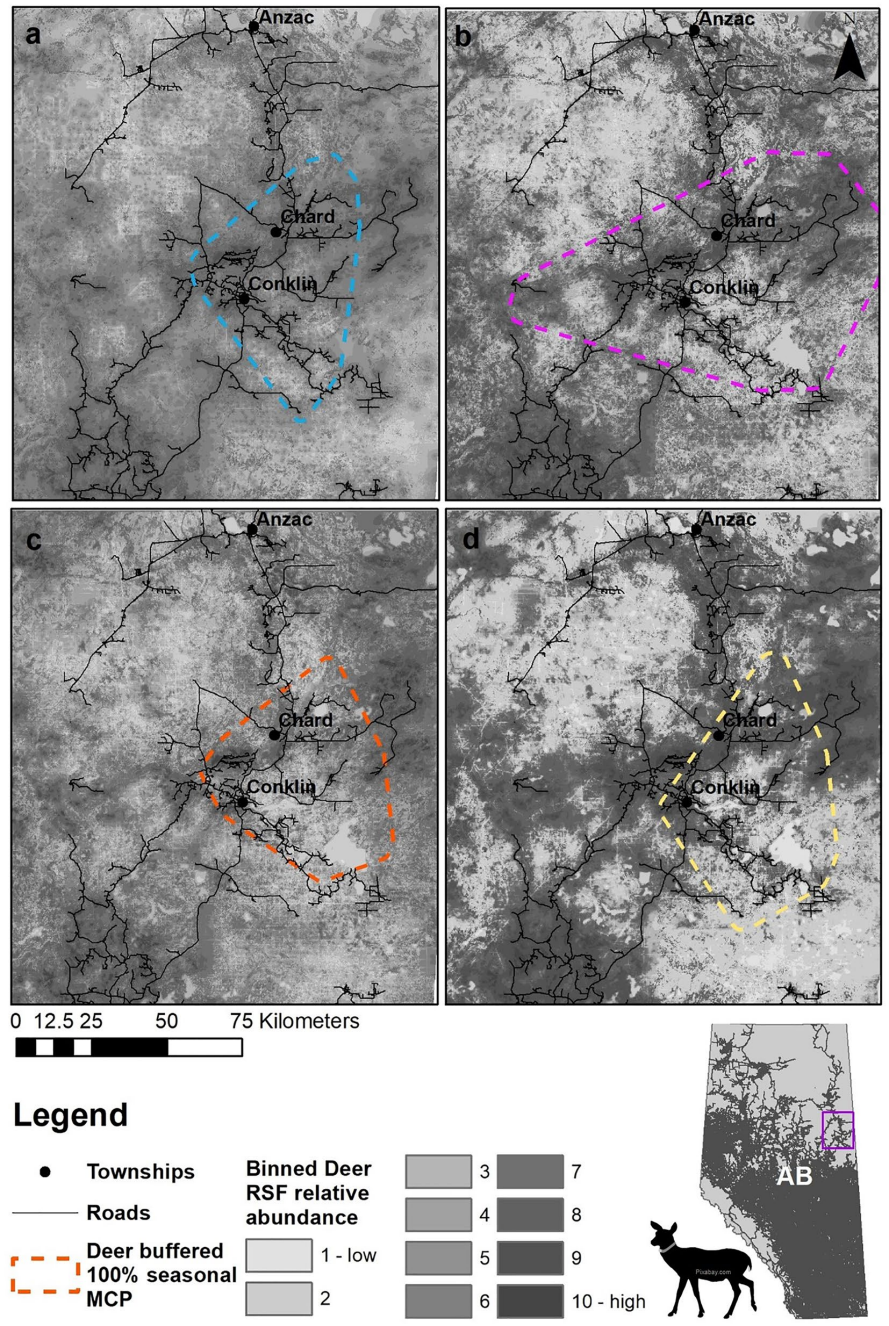
Binned seasonal white-tailed deer population level Resource Selection Function (RSF) relative abundance estimates near Conklin, Alberta Canada with overlaid road network and buffered 100% MCP for (**a**) Winter (cyan) (**b**) Parturition (magenta) (**c**) Summer (red) and (**d**) Rut (yellow) for combined years from 2012 to 2014. Data are spatially extrapolated to extend < 50 km from MCP boundaries.

**Figure 3:**
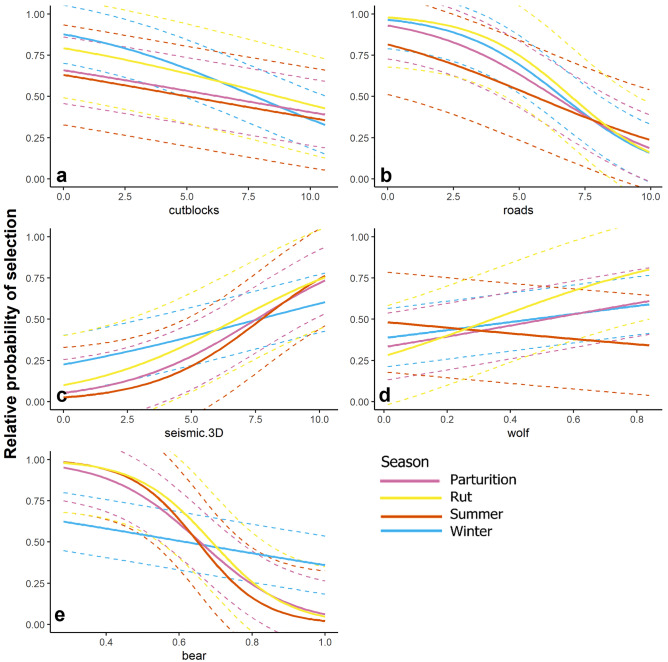
Population-level relative probability of selection (mean and 95% confidence intervals) for white-tailed deer in northeastern Alberta as a function of (**a**) log-transformed distance to cutblocks, (**b**) log-transformed distance to roads, (**c**) log-transformed distance to 3D seismic lines and (**d**) relative probability of wolf occurrence and (**e**) relative probability of black bear occurrence. Confidence intervals incorporate error from the random effect of individual-year.

Data strongly supported the cumulative effects hypothesis. Deer habitat selection was best predicted by a combination of anthropogenic linear and polygonal features, natural habitat composition, and predation risk, across all seasons (Table 3, Fig. 2). Human footprint was the second-best model across all seasons, and resource subsidies outperformed linear features and predation risk during the summer and rut (Table 3). The strength of deer selection and avoidance of landscape features varied across seasons, although the direction of selection remained constant for the majority of covariates (Fig. 3) and supported the importance of forage acquisition at both polygonal and linear anthropogenic features.

**Table 3:** Akaike Information Criteria correction (AICc) results, including change in AICc and corresponding AICc weight, for each model during each of the four deer seasons: winter, parturition, summer and rut.

Season	Model	AICc	∆ AIC	Relative likelihood	AICc weight	Spearman *s*
Winter	Predation	125,931	22,923	0	0	0.573
	Natural	117,657	14,649	0	0	0.958
	Resource	116,049	13,041	0	0	0.920
	Linear	112,488	9480	0	0	0.893
	HF	108,610	5602	0	0	0.938
	Cumulative	103,008	0	1	1	0.975
Parturition	Predation	68,005	20,807	0	0	0.870
	Natural	60,278	13,080	0	0	0.921
	Resource	60,210	13,012	0	0	0.968
	Linear	59,810	12,612	0	0	0.944
	HF	55,291	8093	0	0	0.954
	Cumulative	47,198	0	1	1	0.930
Summer	Predation	26,348	9489	0	0	0.645
	Natural	23,525	6666	0	0	0.901
	Linear	21,909	5050	0	0	0.916
	Resource	20,118	3259	0	0	0.870
	HF	19,010	2151	0	0	0.897
	Cumulative	16,859	0	1	1	0.906
Rut	Predation	36,576	11,785	0	0	0.802
	Natural	35,005	10,214	0	0	0.947
	Linear	30,846	6055	0	0	0.928
	Resource	30,749	5958	0	0	0.928
	HF	28,125	3334	0	0	0.923
	Cumulative	24,791	0	1	1	0.843

Spearman s is the mean value based on k-fold cross validation where k = 10.

**Figure 4:**
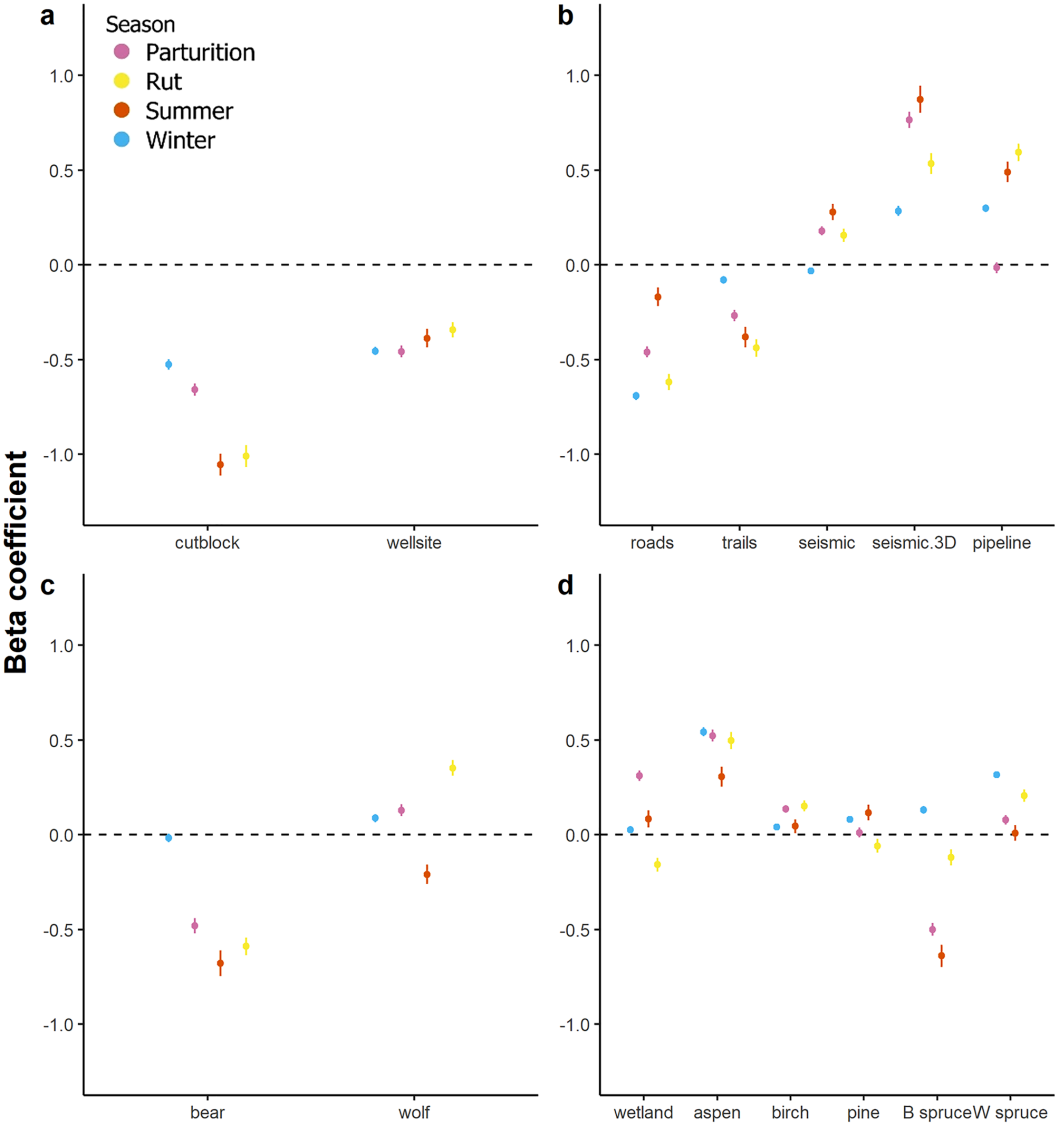
Beta coefficient estimates and 95% confidence intervals from Generalized Linear Mixed-effect Models (GLMMs) for (**a**) polygonal features (**b**) linear features, (**c**) predation risk and (**d**) natural habitat features, for each season: winter, parturition (red), summer (green) and rut (orange). In panels a, b, and c where variables are a measure of distance, negative beta values represent selection and positive values represent avoidance to a feature. Vegetation and predator variables are interpreted as positive for selection and negative for avoidance.

Deer strongly selected areas closer to industrial polygonal features (cutblocks and wellsites, Fig. 4a). Selection for cutblocks was weaker in the winter (β_cutblock_ = − 0.53, 95% confidence interval (CI) = − 0.55 to − 0.50) and parturition seasons (β_cutblock_ = − 0.65, CI = − 0.69 to − 0.63), compared to selection in the rut (β_cutblock_ = − 1.01, CI = − 0.1.07 to − 0.95) and summer (β_cutblock_ = − 1.06, CI = − 1.11 to − 1.00; Fig. 3a, 4a). Wellsite selection was relatively constant across all seasons (Fig. 4a). Deer selected roads and trails, while avoiding seismic lines and pipelines most of the year (Fig. 4b) except winter, although selection was extremely weak (β_seismic_ = − 0.034, CI = − 0.049 to − 0.018); confidence intervals overlapped zero for pipelines during parturition (β_pipeline_ = − 0.016, CI = − 0.045 to 0.013; Fig. 4b). Deer avoided 3D seismic lines more strongly than all other linear features, but less so in winter (β_3D seismic_ = 0.28, CI = 0.26 to 0.31; Fig. 4b). Trails were selected in summer (β_trails_ = − 0.38, CI = − 0.43 to − 0.33) and rut (β_trails_ = − 0.44, CI = − 0.48 to − 0.39), less so in parturition (β_trails_ = − 0.27, CI = − 0.30 to − 0.24), and selection was almost neutral in the winter (β_trails_ = − 0.08, CI = − 0.10 to − 0.06; Fig. 4b). Roads were strongly selected in all seasons (Fig. 4b) though less so in summer (β_roads_ = − 0.17, CI = − 0.22 to − 0.12).

Deer responded differently to areas occupied by black bears compared to wolves. Deer avoided areas with higher occurrence probability of black bears; as expected this signal was weakest in winter (β_bear_ = − 0.018, CI = − 0.040 to − 0.0038; Fig. 4c) when black bears hibernate, lending confidence to our analysis. In contrast there was little consistent response that deer could avoid risky places with higher wolf occurrence. Contrary to expectations deer selected areas of high wolf occurrence during the rut (β_wolf_ = 0.35, CI = 0.31–0.39), weakly selected areas with higher wolf probability of occurrence during winter (β_wolf_ = 0.087, CI = 0.066–0.11) and parturition (β_wolf_ = 0.13, CI = 0.10–0.16), but avoided wolf areas during the summer (β_wolf_ = − 0.21, CI = − 0.26 to − 0.16.

Lastly, the strongest natural habitat drivers of deer selection were aspen (positive, Fig. 4d) and black spruce (negative, apart from winter; β_black spruce_ = 0.13 CI = 0.11–0.15). In summary, both polygonal and linear anthropogenic features played a strong role in deer habitat selection, with deer selecting early seral forage polygonal features, and selecting some linear features while avoiding others. Deer did not avoid areas of high predicted wolf predation risk, and in fact selected areas of estimated frequent wolf occurrence.

## Discussion

Invading boreal white-tailed deer selected habitats with a combination of high-density polygonal and linear features, interspersed within natural landcover, suggesting forage acquisition drove the most consistent and greatest effect sizes in a model assessing both forage and predation. The boreal forest is changing in landscape composition and ecological community structure as the climate warms and resource extraction increases^59,60^. The twenty-first century boreal landscape differs from any historical form^60^, and anthropogenic features have the potential to shape the biotic processes within, including species’ range expansion and invasion. Our results suggest that these features may be contributing to Nearctic range expansion in white-tailed deer in all seasons.

Linear features had the strongest effect on deer habitat selection in the winter and parturition. Industrial linear features are pervasive in the boreal landscape^17^, and we hypothesized that deer would perceive these features as risky, due to their frequent use by apex predators such as wolves^26,29^. Deer avoided 3D seismic and pipelines, while selecting roads and trails throughout the year. Selection for these larger linear features may be explained by early seral vegetation forage subsidies at roadsides and verges and through “edge effects” into forest interiors^30,61^. Deer selected areas closer to roads and trails relative to the available landscape, likely due to an increase in these edge effects. The direction and magnitude of selection may be mediated by perceived risk of predation: features selected by deer are not only expected to have forage subsidies, but also are associated with human activity which may shield deer from predators^62^. However, deer selected areas farther from 3D seismic lines (generally without much early seral forage) and pipelines, which are less associated with predictable human activity and this behaviour could be inferred as predation avoidance.

Deer also selected areas closer to polygonal block features, particularly during the summer and rut where the resource subsidy hypothesis outcompeted linear features. The addition of both block features and linear features to predation risk and natural heterogeneity better explained deer selection than any set of features alone. The attraction to polygonal and large linear features is problematic as both features continue to expand as demand for oil increases^28,63,64^. The continuation of deforestation and linearization of the Nearctic boreal forest for seismic exploration, as well as ongoing timber harvesting, will likely sustain and facilitate boreal deer expansion.

Deer selection of habitat features associated with higher predicted predation risk by wolves or bears varied seasonally. During winter and parturition, deer weakly selected areas with greater probability of wolf activity, while strongly selecting it in the rut. This could be interpreted as resulting from two processes. First, wolves are cueing in on areas with abundant prey, especially deer: wolf distribution is strongly and positively associated with moose and deer^5,19^. Second, deer are either unable to avoid areas with wolves, or do not prioritize risk avoidance, instead prioritizing features that offer abundant resources despite the increased likelihood of encounter with wolves. Deer displayed the weakest association with wolves during the summer and we infer deer change their behaviour to prioritize actively avoiding encounters with wolves during this time when forage is abundant, and fawns are mobile but still vulnerable. Moreover, deer exhibited the weakest predator avoidance behaviour (in this case, strong positive association) during the rut, when mating occurs^20^. A reduction in vigilance behaviour during the rut was expected, due to deer prioritizing mating and forage acquisition to meet energetic requirements of reproduction and withstanding forage-limiting winter conditions^65,66^. Deer avoided habitat associated with bears during all seasons when bears are active on the landscape, suggesting spatial anti-predator response. Additionally, black bears have omnivorous diets, and opportunistically depredate fawns of deer and other neonates of boreal Cervidae^47^. We can therefore not expect bears to display the same increase in activity in deer habitat as we do with wolves. Alternatively, some habitat features avoided by bears are highly selected by deer, such as upland and lowland deciduous forest. Therefore, this relationship may arise due to an incompatibility of resource quality rather than being an example of avoidance behaviour; although bears avoided few features, so we deem this unlikely.

Our measure of predation risk across space (risky places) was the probability of predator occurrence based on monthly predator detections at camera sites over a three-year period^5^. We were conservative in that our estimates were extrapolated no farther than 50 km from the camera sites, and landscape features were categorized using regression coefficient estimates at the same spatial scale as they were measured in the best-fit models^5^. Nevertheless, it is possible that the wolf risk map did a poor job of predicting true wolf occurrence, manifesting as a positive relationship between wolves and deer. For example, due to limitations with sample size, we were unable to account for changes in predator distributions across deer biological seasons. Future research could include direct measures of predation risk (e.g. collared predators, deer mortalities) to better understand the extent to which deer make energetic trade-offs in the presence of predators, further exploring the risky times *v*s. risky places hypotheses^67^. However, a multi-species telemetry-based movement ecology study is needed for such high-resolution data. In the absence of this expensive research, camera traps can provide a useful index of predator occurrence for spatial inferences, and we hope camera data will inform analyses to greater extent in the future^68^.

Anthropogenic landscape change is a leading driver of seasonal deer habitat selection behaviour. Landscape change is also associated with deer distribution and reproduction at landscape scales^27,35^, and given these effects are consistent across the ecological hierarchy (behaviour, distribution, populations), we infer it is a key driver of deer expansion in western boreal landscapes. Range-expanding deer in the Nearctic boreal forest have numerous ecological implications for native fauna and flora. Deer are apparent competitors with declining woodland caribou^24^—where increasing deer populations have increased wolf populations, driving down caribou^26^. The recovery of woodland caribou is dependent on the reduction of habitat loss within their ranges and predation pressure by grey wolves^69^, and hence management of invasive deer. Local increases in deer populations can alter ecosystems by over-browsing, which reduces plant growth, forest understorey diversity, and consequently animal diversity^20,70^. Estimates for deer abundance are difficult to ascertain across terrain and cover types^71,72^ and whether carrying capacity for deer has been met in this system is not known. Anthropogenic landscape change facilitating deer invasion may entrain future landscape and biotic change where deer populations are on the rise.

Mitigating the multiple sources of anthropogenic resource subsidies for deer through landscape restoration may eliminate forage subsidies and reduce their ability to persist in harsh winter conditions – and hence curtail their expansion and ancillary biotic changes to the boreal ecosystem. Seismic line restoration, implemented where slow natural regeneration rates and rapid development of new seismic lines call for silvicultural intervention^28^, is already underway. The current caribou recovery plan for Alberta lists 10,000 km of restored seismic lines as a primary objective in the recovery of declining herds^73^ and contemporary research suggests there may be some effect on deer^74^. However, restoration of polygonal features is also needed to return the boreal landscape to one largely lacking sufficient forage suitable for deer range expansion.

As global needs for food, wood, minerals and petroleum increase, landscapes are reshaped and biodiversity declines^75^. The Canadian boreal forest is a substantial source of wood^18^ and energy^76^ while also serving a key carbon sink to mitigate climate change^77^. As human footprint grows and diversifies, the effects of this reshaped landscape on biota serve as an early model for boreal communities facing environmental change from resource extraction.

## Supplementary Information

Supplementary Information.

## Data Availability

Seasonal deer data will be available on Dryad.
