# Supplementary material for: Cumulative effects of human footprint, natural features and predation risk best predict seasonal resource selection by white-tailed deer
Source: Sci Rep. 2022 Jan 20;12:1072. doi: 10.1038/s41598-022-05018-z (PMC8776810; doi:10.1038/s41598-022-05018-z)
Supplement: Supplementary file 1 — Supplementary Information. [file 41598_2022_5018_MOESM1_ESM.pdf]

Supplementary Material

Title: Cumulative effects of human footprint, natural features and predation risk best predict seasonal resource selection by white-tailed deer

Authors: Siobhan Darlington[1], Andrew Ladle[2], A. Cole Burton[3], John P. Volpe[2], & Jason T. Fisher[2]

[1] Department of Biology, University of British Columbia Okanagan Campus, 1177 Research Road, Kelowna, BC Canada V1V 1V7

[2] School of Environmental Studies, University of Victoria PO Box 1700 STN CSC, Victoria BC V8W 2Y2 Canada.

[3] Department of Forest Resources Management, University of British Columbia, Forest Sciences Centre 2045 – 2424 Main Mall, Vancouver BC V6T 1Z4 Canada.

Corresponding Author: Andrew Ladle

E-mail: aladle@uvic.ca

Phone: 1-778-700-7437

Table S1. Top models and associated beta coefficient estimates, with standard errors (SE) for wolf (n = 2,508 total detections) and black bear (n = 2,657 total detections) were used in the development of GLM models for monthly occurrence frequency from Fisher & Burton (2018). These values were used to extrapolate annual predation risk for each predator species in ArcMap 10.5.

Species	Scale of analysis	Covariate	Estimate	SE
Wolf	2250m	Intercept	-1.75	0.07
		Block features	0.36	0.08
		Trails	0.35	0.08
		Upland spruce	0.18	0.08
		Upland deciduous	0.17	0.07
		Water	0.14	0.07
		Open wetland	0.12	0.07
		Lowland deciduous	0.12	0.07
		Roads	-0.26	0.09
Black bear	500m	Intercept	-1.18	0.07
		Water	0.15	0.07
		Upland shrubs	-0.11	0.07
		Roads	-0.11	0.08
		Lowland deciduous	-0.14	0.07
		Block features	-0.24	0.09
		3D seismic lines	-0.44	0.08

Table S2. Cumulative effects model estimated beta coefficients and associated lower (LCI) and upper (UCI) confidence intervals.

	β Coefficient	LCI	UCI
Winter RSF			
Intercept	-0.064	-0.217	0.089
wolf	0.087	0.066	0.107
bear	-0.018	-0.040	0.004
aspen	0.542	0.520	0.565
birch	0.041	0.025	0.058
pine	0.081	0.065	0.098
B spruce	0.131	0.111	0.151
W spruce	0.317	0.301	0.333
wetland	0.026	0.007	0.044
roads	-0.692	-0.713	-0.671
seismic	-0.034	-0.049	-0.018
seismic.3D	0.284	0.259	0.310
pipeline	0.298	0.278	0.319
trails	-0.080	-0.100	-0.059
cutblock	-0.526	-0.553	-0.498
wellsite	-0.456	-0.477	-0.435
Parturition RSF			
Intercept	-0.144	-0.376	0.088
wolf	0.129	0.098	0.159
bear	-0.481	-0.520	-0.442
aspen	0.522	0.492	0.553
birch	0.136	0.116	0.156
pine	0.011	-0.015	0.038
B spruce	-0.500	-0.533	-0.467
W spruce	0.079	0.054	0.103
wetland	0.311	0.285	0.337
roads	-0.460	-0.488	-0.432
seismic	0.179	0.155	0.204
seismic.3D	0.765	0.723	0.807
pipeline	-0.016	-0.045	0.013
trails	-0.268	-0.297	-0.239
cutblock	-0.659	-0.691	-0.626
wellsite	-0.457	-0.489	-0.426
Summer RSF			
Intercept	-0.361	-0.773	0.051
wolf	-0.210	-0.260	-0.159
bear	-0.679	-0.744	-0.613
aspen	0.307	0.256	0.359
birch	0.044	0.008	0.080
pine	0.116	0.076	0.156
B spruce	-0.640	-0.697	-0.582
W spruce	0.009	-0.031	0.049
wetland	0.083	0.040	0.127

roads	-0.169	-0.216	-0.122
seismic	0.279	0.238	0.321
seismic.3D	0.873	0.803	0.944
pipeline	0.490	0.437	0.543
trails	-0.382	-0.434	-0.329
cutblock	-1.055	-1.111	-0.998
wellsite	-0.387	-0.435	-0.340
Rut RSF			
Intercept	0.149	-0.239	0.537
wolf	0.352	0.311	0.394
bear	-0.589	-0.635	-0.544
aspen	0.497	0.453	0.541
birch	0.152	0.124	0.180
pine	-0.059	-0.095	-0.024
B spruce	-0.120	-0.162	-0.078
W spruce	0.206	0.175	0.237
wetland	-0.159	-0.195	-0.122
roads	-0.618	-0.660	-0.577
seismic	0.156	0.121	0.191
seismic.3D	0.534	0.480	0.588
pipeline	0.594	0.548	0.639
trails	-0.439	-0.484	-0.393
cutblock	-1.009	-1.065	-0.954
wellsite	-0.343	-0.383	-0.303

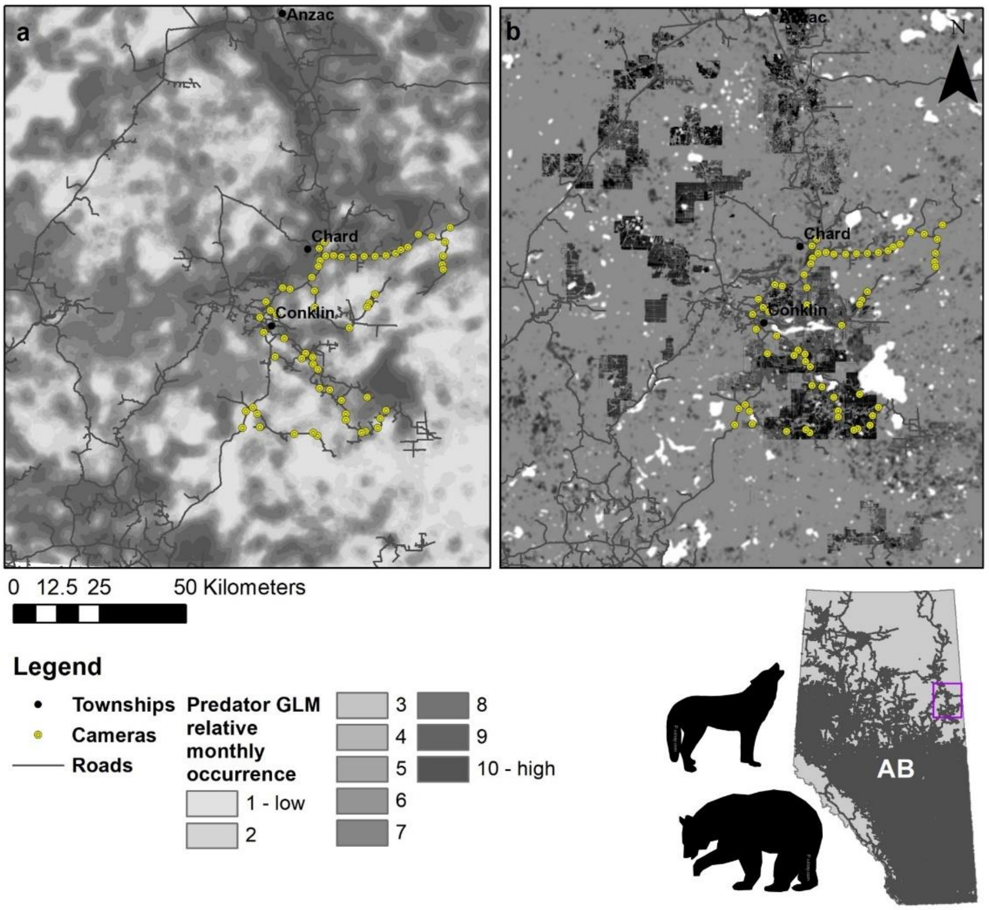

Figure S1. Mapped (a) wolf and (b) black bear relative distribution probability were significantly correlated with raw monthly camera detections from the original models by Fisher & Burton (2018) (Table S1). Wolf relative distribution probability was notably higher in areas with observed deer locations, indicating wolves overlapped spatially with deer. Black bears spanned the entire study area in low densities, responding (negatively) to 3D seismic lines. Consequently, deer used habitat with higher (inferred) wolf predation risk than other available space in all seasons and used habitat with lower black bear predation risk than available in all seasons except winter (when bears hibernate).

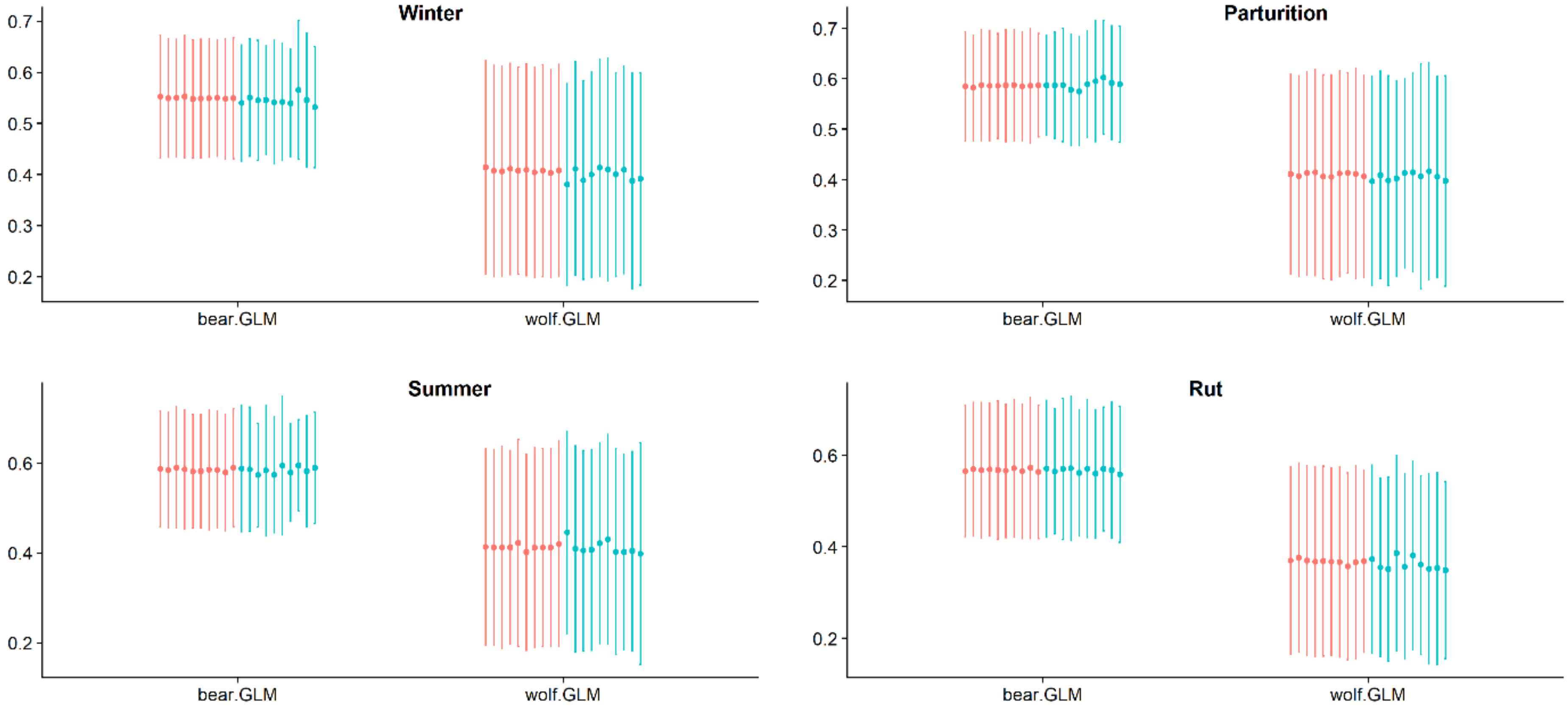

Figure S2. Bootstrapped available distributions (mean ± standard deviation) for individuals with largest (red) and smallest (blue) sample sizes for bear and wolf variables for each season.

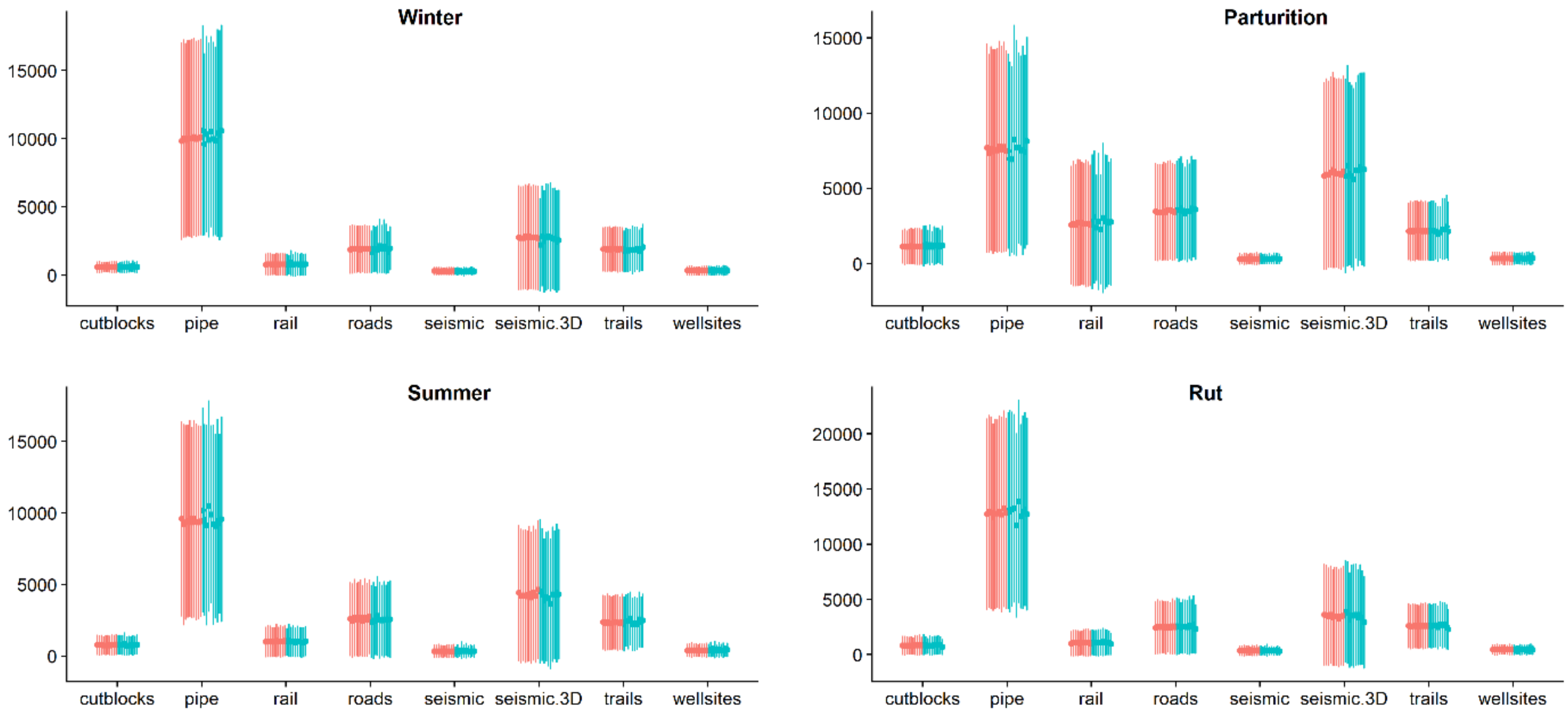

Figure S3. Bootstrapped available distributions (mean ± standard deviation) for individuals with largest (red) and smallest (blue) sample sizes for distance variables for each season.

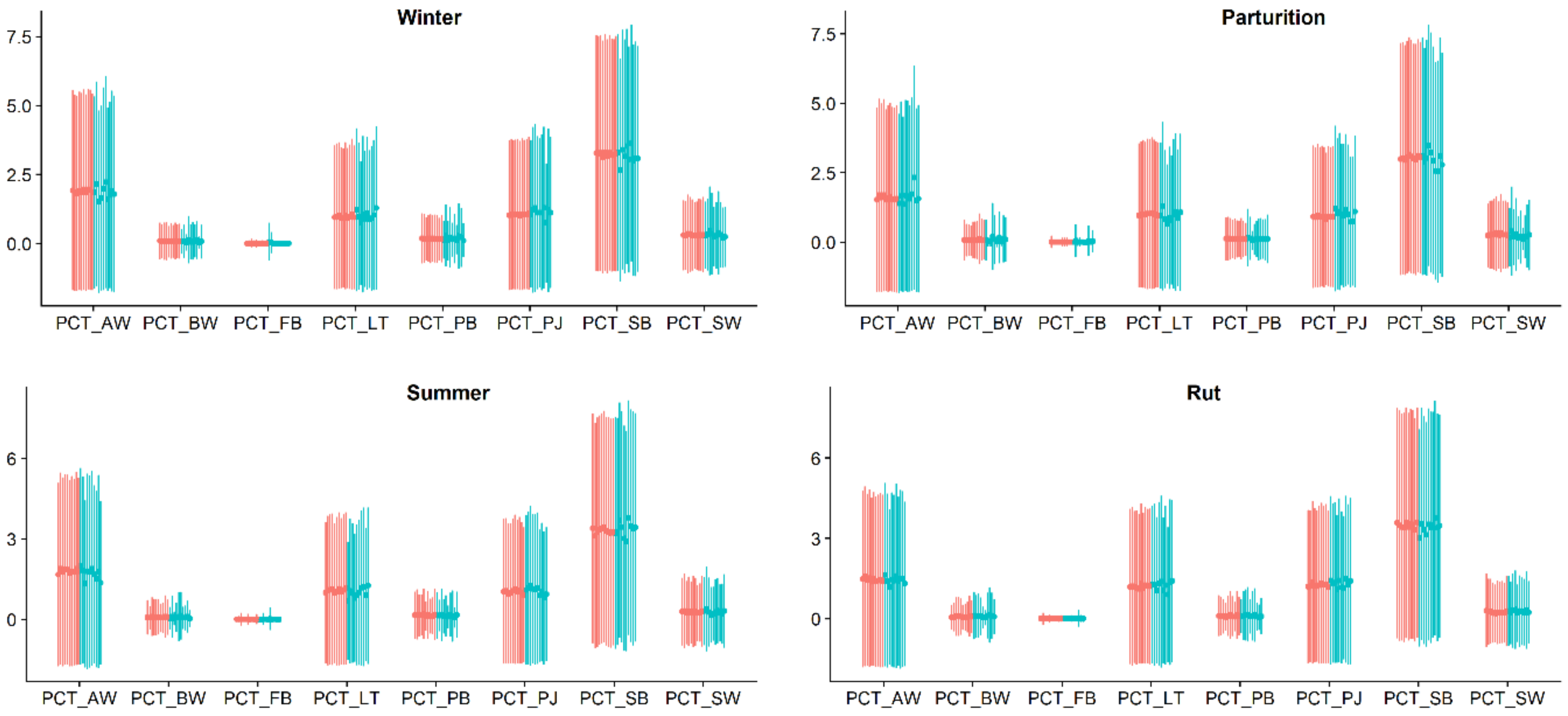

Figure S4. Bootstrapped available distributions (mean ± standard deviation) for individuals with largest (red) and smallest (blue) sample sizes for natural vegetation variables for each season.
